# A DNA barcode reference library of Neuroptera (Insecta, Neuropterida) from Beijing

**DOI:** 10.3897/zookeys.807.29430

**Published:** 2018-12-17

**Authors:** Pan Yi, Pei Yu, Jingyi Liu, Huan Xu, Xingyue Liu

**Affiliations:** 1 Department of Entomology, China Agricultural University, Beijing 100193, China China Agricultural University Beijing China; 2 Department of Biology, Tokyo Metropolitan University, Tokyo 192-0397, Japan Tokyo Metropolitan University Tokyo Japan

**Keywords:** China, cytochrome c oxidase subunit I, mitochondrial DNA, lacewings, taxonomy

## Abstract

Neuroptera (lacewings) is one of the ancient holometabolous insect groups, but some extant species stand as important natural enemies for biological control. As the capital city of China, Beijing has a rich fauna of Neuroptera, previously with 47 species recorded and sorted in 32 genera of seven families. In this study, DNA barcoding based on sequences of COI gene fragments is used to discriminate lacewing species from Beijing. 217 DNA barcode sequences belonging to 49 species were successfully obtained. The COI barcode data worked well for identification of almost all lacewing species herein examined except *Pseudomalladaprasinus* (Burmeister), in which cryptic species may exist. Twenty species of Neuroptera are newly recorded from Beijing. Besides, Nothochrysinae is first recorded from Beijing. *Chrysopidiaciliata* (Wesmael) and *Drepanepteryxalgida* (Erichson) are first recorded from China.

## Introduction

Neuroptera (lacewings) is the most species-rich order of the superorder Neuropterida. Hitherto, there are about 6000 described species worldwide in 16 families (Engel et al. 2018; Winterton et al. 2018). Adult lacewings in general are delicate insects, having two pairs of membranous wings with highly reticulate venation, while the lacewing larvae are characterized by the specialized mandibles and maxillae that are combined into a pair of sucking jaws. The common groups of Neuroptera consist of Chrysopidae (green lacewings), Hemerobiidae (brown lacewings), Myrmeleontidae (antlions), and Coniopterygidae (dusty lacewings), while the other lacewing families each comprises much fewer species and some of these families (e.g., Nevrorthidae, Rhachiberothidae, Ithonidae, Psychopsidae) have much narrower distributions. However, the diversification of Neuroptera in morphology as well as in biology is remarkable (Aspöck et al. 2012; Engel et al. 2018).

Because of the predatory feeding habits, some lacewing species, e.g., the species of Chrysopidae, Hemerobiidae, and Coniopterygidae, are economically important and have been used for the biocontrol of agricultural pest insects (Goolsby et al. 2000; McEwen et al. 2001; Sato and Takada 2004; Bezerra et al. 2006; Abdrabou 2008; Vidya et al. 2010; Messelink et al. 2016). However, the species identification of these lacewing groups is not easy to handle, particularly for people who are not the specialists of Neuroptera, because there are many morphologically similar species, which require examination of detail morphological characters, such as marking patterns on body and genitalia. Moreover, for some species-rich groups, such as Chrysopidae, the taxonomy still requires comprehensive revision (Henry and Wells 2010, Henry et al. 2013, 2014; Duelli et al. 2016; Dai et al. 2017).

DNA barcoding has become the most popular approach for the species identification and the assignment of specimens throughout all life stages to described species (Hebert et al. 2003a, b). In animals, including insects, an app. 660 base pair (bp) fragment of the mitochondrial cytochrome *c* oxidase subunit I (COI) gene has been chosen as standardized barcode marker (Hebert et al. 2003a, b). As a molecular marker for efficient species identification, DNA barcoding with COI yields excellent results across a broad spectrum of insects, and even reveals unknown cryptic species diversity of certain groups (Smith et al. 2006; Burns et al. 2008; Huemer et al. 2014; Song et al. 2018). Besides, DNA barcoding based on COI with the Automatic Barcode Gap Discovery and the Bayesian Poisson Tree Processes model was also used to separate some new antlion species (Pantaleoni and Badano 2012; Badano et al. 2016). Notwithstanding, there is still limited number of works on DNA barcoding of Neuroptera (Morales and Freitas 2010; Morinière et al. 2014; Choi et al. 2015; Price et al. 2015).

Beijing, as the capital city of China, is located at northern China and surrounded by Hebei Province, belonging to the eastern Palaearctic region. To the west of Beijing is Mt. Xishan, forming the eastern flank of the Taihang Mountains range, which runs north-south up the spine of Hebei province. Mt. Xishan covers nearly all of Fangshan and Mentougou Districts west of the city. The mountains north of Beijing including Mt. Wulingshan, Mt. Jundushan, and Mt. Fenghuanling all belong to the Yanshan range, which runs east-west, across northern Hebei Province. Climate of Beijing is typical humid continental monsoon climate with hot and rainy summers, cold and dry winters. The majority flora of Beijing is temperate deciduous forest. Despite high-speed increase of economic development and population, relatively well-preserved natural environment still remains in Beijing, particularly in the aforementioned mountainous areas.

Concerning Neuroptera, Beijing has relatively rich fauna of lacewing species, currently with 47 species recorded based on the recently published catalogue of the Chinese Neuropterida (Oswald 2018; Yang et al. 2018). Remarkably, the lacewing fauna of Beijing appears still not to be thoroughly explored considering recent findings of new species from this area (Zhao et al. 2013; Zhang et al. 2014).

Here we present a preliminary DNA barcode library for the lacewing species from Beijing. A total of 217 barcode sequences were amplified, and this dataset comprises the barcodes of 49 species (including seven undetermined species). Twenty species are newly recorded from Beijing, and two of them are first recorded from China (Figures 2–4; Suppl. material 1–3: Figures S1–3). An updated checklist of species of Neuroptera from Beijing is provided (Suppl. material 6: File S2).

## Material and methods

### Sampling of specimens

The lacewing specimens herein studied were collected between 2013 and 2017 using sweeping net and light trap. The collecting areas mainly comprise the Xiaolongmen Forestry Park, Mentougou District, northwestern Beijing, the Wulingshan National Nature Reserve that is located across Miyun District in northeastern Beijing and Xinglong County in Hebei Province, an organic orchard in Wangjiayuan Village, Changping District, northern Beijing, and the Olympic Forest Park, Chaoyang District in the metropolitan area of Beijing. The specimens were preserved in ethanol (95%) and identified based on the morphological characteristics using the keys to the species (Aspöck et al. 1980; Liu 2003; Yang et al. 2005; Zhao 2016; Wang et al. 2018). The number of specimens per species ranged from 1 to 26. All specimens herein studied are deposited in the Entomological Museum of China Agricultural University (CAU), Beijing, China.

### DNA extraction

Total genomic DNA was isolated from mid legs using the TIANamp Genomic DNA Kit (TIANGEN Inc., Beijing, China) according to the manufacturer’s instructions. The barcoding fragments of COI were amplified by Polymerase chain reactions (PCR). The reaction was conducted in a final volume of 25 μL consisting of 14.5 μL of ddH_2_O, 1 μL (10 μM) of each of the primers, 2 μL of dNTP, 0.5 μL of polymerase and 1 μL DNA template (~30 ng). For Chrysopidae, the COI gene fragments were amplified with specific primers, i.e., COIa–F (5’–TACAATTTATCGCCTAAACTTCAGCC–3’) and COIa–R (5’–CCCGGTAAAATTAAAATATAAACTTC–3’) because the universal primers (i.e., LCO1490 and HCO2198; see Folmer et al. 1994) did not work well for this group in our study. For the other groups, the COI gene fragments were amplified with the aforementioned universal primers, i.e., LCO1490 (5’–GGTCAACAAATCATAAAGATATTGG–3’) and HCO2198 (5’–TAAACTTCAGGGTGACCAAAAAATCA–3’). The PCR amplifications were run under the following conditions: initial denaturation at 95 °C for a half minute, followed by 40 cycles of 10 seconds at 95 °C, 50 seconds at 47 °C, and 2 minutes at 65 °C; a final extension phase of 65 °C for 10 minutes. The PCR products were subjected to electrophoresis in 1% agarose gel and stained with GoldView (1ng/mL) to confirm amplification. Amplicons were sequenced bidirectionally, using the BigDye® Terminator v3.1 Cycle Sequencing Kit (Applied Biosystems, Foster City, CA, USA) on an ABI 3730XL Genetic Analyzer (PE Applied Biosystems, San Francisco, California, USA).

### Data analysis

The final consensus COI sequences were obtained after overlapping both forward and reverse sequences by ContigExpress. All sequence data are deposited in GenBank (see Accession number in File S1). All sequences were aligned using Clustal W (Thompson et al. 1994) and analyzed using a neighbor-joining cluster analysis (NJ; Saitou and Nei. 1987) based on the Kimura-2-Parameter (K2P; Kimura 1980) distances with MEGA v. 5.0 (Tamura et al. 2011). The consequence of NJ tree was explored the Newick tree file and subsequently modified with FigTree v1.4.3. (http://tree.bio.ed.ac.uk/software/figtree/, Andrew 2006). Nucleotide composition and the K2P distances between and within species were also calculated by MEGA v. 5.0. Additional species-delimitation methods were also included in our study, i.e., the Automatic Barcode Gap Discovery (ABGD; Puillandre et al. 2012) and the Bayesian Poisson Tree Processes model (bPTP; Zhang et al. 2013). ABGD is an automatic procedure that sorts the sequences into hypothetical species based on the threshold of pairwise genetic distances. The ABGD analyses were performed on the web interface (http://wwwabi.snv.jussieu.fr/public/abgd/). The K2P distance was selected for the datasets, and other parameters were set to default except the default values of steps=50 and relative gap width (X)=0.5. bPTP is an updated version of the original PTP with bayesian posterior probability, providing more accurate results, maximal likelihood solution and bayesian supported solution, for species delimitation i.e., bPTP_ML and bPTP_BS. For the bPTP analyses, the ML trees were constructed using RAxML v8.2.10 under the GTRGAMMA evolutionary model and performed on the bPTP web server (http://species.h-its.org/), with 0.25 burn-in and 500,000 MCMC generations. To test the reliability of results, each run was checked for convergence by visualizing the likelihood plot. The COI sequence of *Lepicerusinaequalis* (Coleoptera: Lepiceridae; GenBank: KJ871320) and *Nebriaformosana* (Coleoptera: Carabidae; GenBank: KT306091) were selected as outgroups because of the close relationship between Coleoptera and Neuropterida (Misof et al. 2014).

## Results

**Figure 1.**
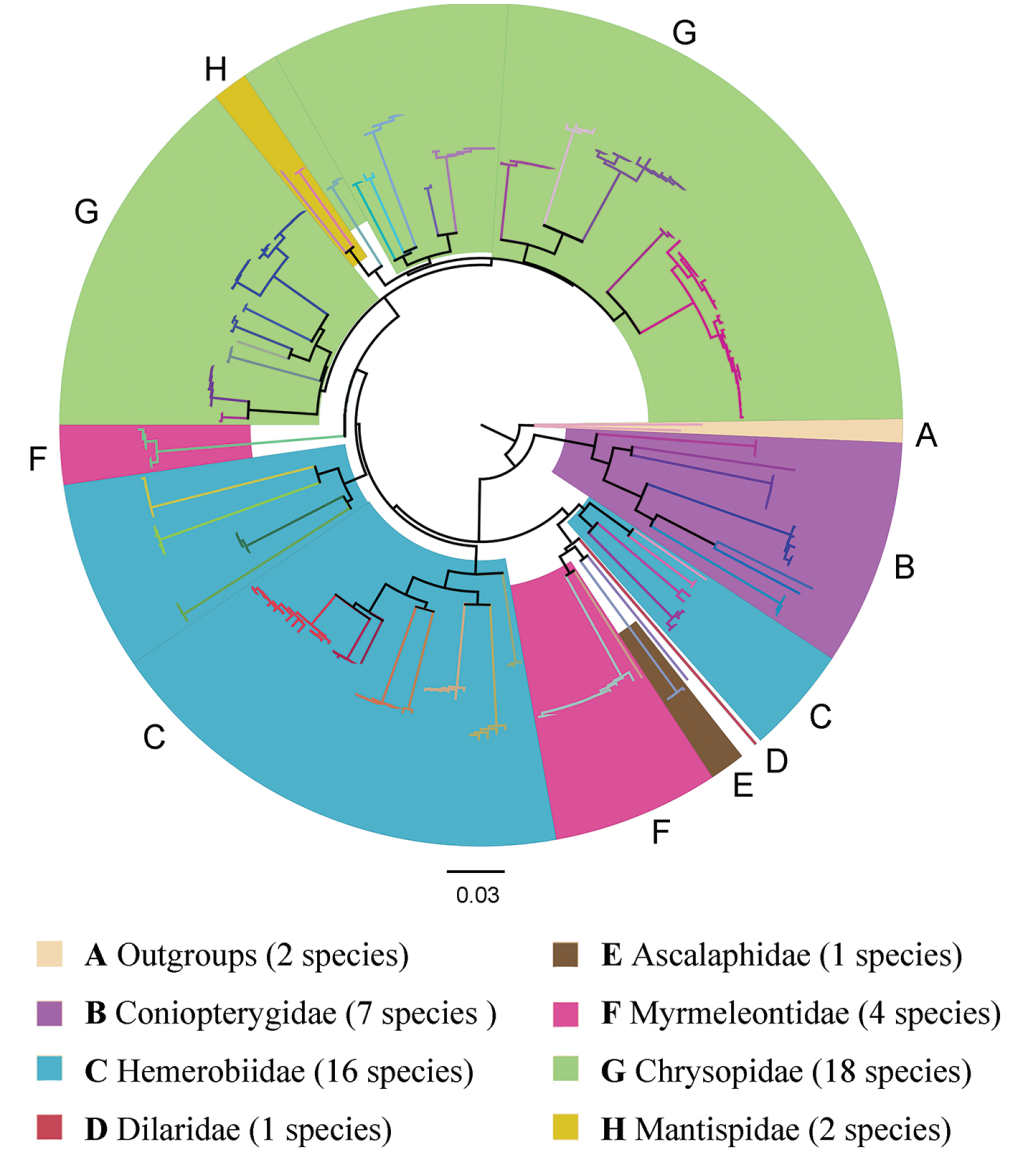
Neighbor-joining tree based on the COI sequence dataset of the lacewing species from Beijing. Different color of clades represents different species.

The present study generated 217 sequences of 639 bp each, with an average nucleotide composition of 39.5% thymine (T), 15.8% cytosine (C), 28.4% adenine (A), and 16.3% guanine (G). Base frequencies analysis revealed low GC-contents (average: 31.1%) for the barcode fragment. The above COI barcode sequences were found to belong to 49 species of Neuroptera. A full list of these species and their collecting information are presented in the Suppl. material 5: File S1. A threshold of the COI genetic distance ≥ 2% was applied for a rough differentiation between intraspecific and interspecific distances based on Hebert et al. (2003b). Intraspecific distances ranged from zero to 2.7% (see *Pseudomalladaprasinus* (Burmeister, 1839); Suppl. material 8: Table S2. Interspecific distances ranged between 2.9% (see species of *Pseudomallada*) and 25.3% (see *Semidalisaleyrodiformis* (Stephens, 1836) and *Coniopteryxplagiotropa* Liu & Yang, 1997; Suppl. material 7: Table S1). The number of recovered clusters (= 49), each of which can be clearly separated from all neighboring species (Figure 1), is identical to the number of species identified based on morphological characters, suggesting that the species in question can be identified unambiguously by DNA barcoding.

### Coniopterygidae

Seven species of Coniopterygidae from Beijing were studied, including two species newly recorded from Beijing, i.e., *Conwentziasinica* Yang, 1974 and *Semidalisbicornis* Liu & Yang, 1993, and two undetermined species of *Coniopteryx* with a minimum mean distance 10.9% (Suppl. material 7: Table S1). *Semidalisaleyrodiformis* and *Coniopteryxplagiotropa* possess a maximum mean distance 23.3%. Results of species delimitation based on ABGD and bPTP_ML are congruent with our identification based on morphology (Figure 5A). However, bPTP_BS divided *Semidalisaleyrdiformis* into five Molecular Operational Taxonomic Units (MOTUs; n = 5) with low posterial probabilities (< 60%). It is probably overestimated because the intraspecific variation within the specimens of *Semidalisaleyrodiformis* is 0.

**Figure 2.**
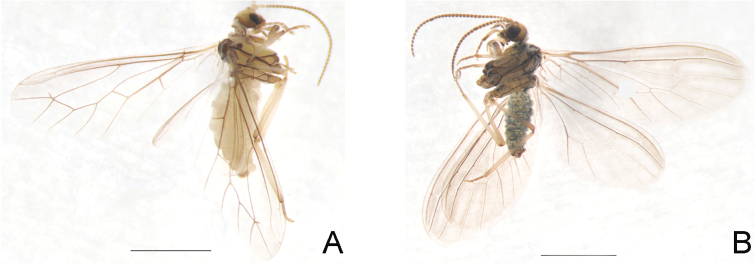
Habitus photographs of species of Coniopterygidae newly recorded from Beijing. **A***Conwentziasinica* Yang, 1974 **B***Semidalisbicornis* Liu & Yang, 1993. Scale bar: 1 mm.

### Chrysopidae

The present analysis resulted in 18 species of Chrysopidae from Beijing. Three of them could not be identified to species. Among them, there are 10 species newly recorded from Beijing, including *Chrysopaintima* McLachlan, 1893, *Chrysoperlafurcifera* (Okamoto, 1914), *Chrysopidiaciliata* (Wesmael, 1841), *Malladaflavimaculus* Yang & Yang, 1991, *Pseudomalladacognatellus* (Okamoto, 1914), *Pseudomalladaprasinus* (Burmeister, 1839), *Pseudomalladaqinlingensis* (Yang & Yang, 1989), *Ninetagrandis* Navás, 1915, *Ninetashaanxiensis* Yang & Yang, 1989 and *Nothochrysasinica* Yang, 1986. Furthermore, *Nothochrysasinica* represents the first record of the subfamily Nothochrysinae from Beijing, while *Chrysopidiaciliata* is first recorded from China.

**Figure 3.**
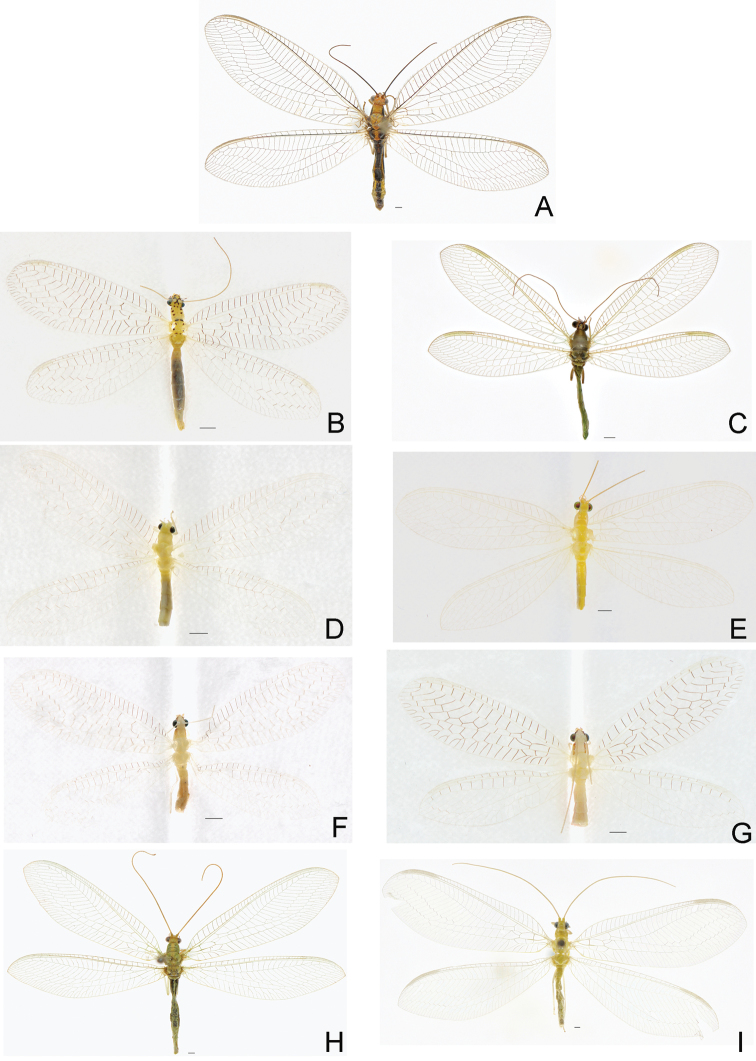
Habitus photographs of species of Chrysopidae newly recorded from Beijing. **A***Nothochrysasinica* Yang, 1986 **B***Chrysopaintima* McLachlan, 1893 **C***Chrysoperlafurcifera* (Okamoto, 1914) **D***Chrysopidiaciliata* (Wesmael, 1841) **E***Malladaflavimaculus* Yang & Yang, 1991 **F***Pseudomalladacognatellus* (Okamoto, 1914) **G***Pseudomalladaqinlingensis* (Yang & Yang, 1989) **H***Ninetagrandis* Navás, 1915 **I***Ninetashaanxiensis* Yang & Yang, 1989. Scale bar: 1 mm.

For testing the present identification, we also compare the barcode sequences of several green lacewing species [i.e., BINS: ACF7085 (*Chrysopaformosa*); AAB0373 (*Chrysoperlanipponensis*); AAJ3493 (*Chrysopidiaciliata*); ABU9179, ACF9046 (*Pseudomalladaprasinus*); GenBank: KJ592516 (*Chrysopapallens*)] obtained from the Barcoding of Life Data systems (BOLD, http://www.barcodinglife.org/) and the National Center Biotechnology Information (NCBI, https://www.ncbi.nlm.nih.gov/) by using a neighbor-joining cluster analysis based on the K2P distances with MEGA v. 5.0. Most of these sequences were respectively clustered with those of same species herein sequenced, verifying our identification (Suppl. material 4: Figure S4). However, in *P.prasinus* specimens from Europe and from Beijing are clearly assigned into two clades. A similar result concerning *P.prasinus* from Europe and East Asia was also found in a phylogenetic analysis of *Pseudomallada* combining morphology, life-history traits, and nuclear DNA sequences (Duelli et al. 2017).

**Figure 4.**
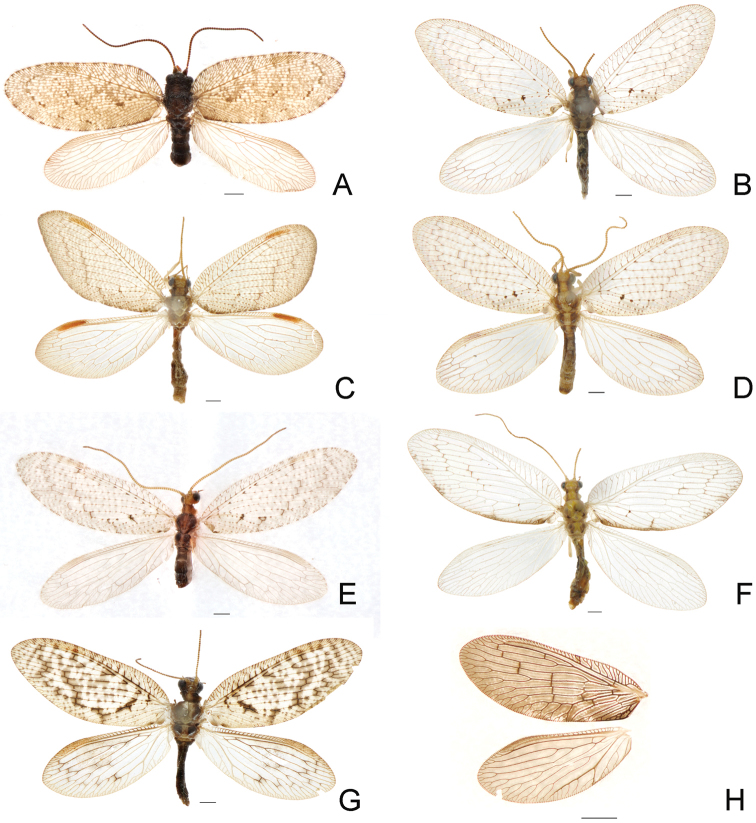
Habitus photographs of species of Hemerobiidae newly recorded from Beijing. **A***Drepanepteryxalgida* (Erichson, 1851) **B***Hemerobiusbispinus* Banks, 1940 **C***Hemerobiusexoterus* Navás, 1936 **D***Hemerobiushumulinus* Linnaeus, 1758 **E***Hemerobiusjaponicus* Nakahara, 1915 **F***Hemerobiusmarginatus* (Stephens, 1836) **G***Hemerobiussubtriangulus* Yang, 1987 **H***Sympherobiusmanchuricus* Nakahara, 1960. Scale bar: 1 mm.

**Figure 5.**
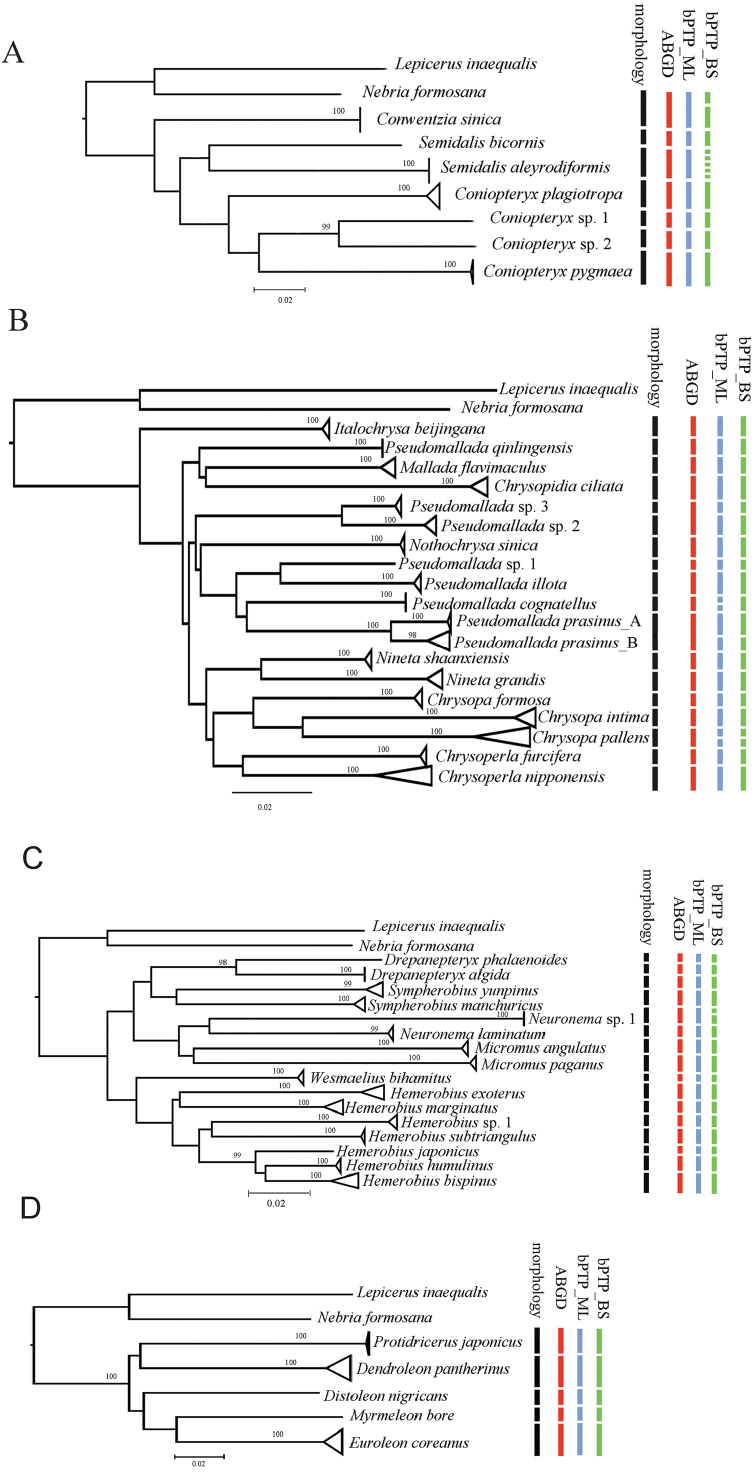
Neighbor-joining tree and result of molecular species delimitation based on COI barcodes. **A**Coniopterygidae**B**Chrysopidae**C**Hemerobiidae**D**Myrmeleontidae and Ascalaphidae. The terminal nodes in the tree are collapsed for each morphological species, the width of triangles shows the sequence divergence. Only bootstrap supports (1,000 replicates) > 0.95 are labelled.

**Figure 6.**
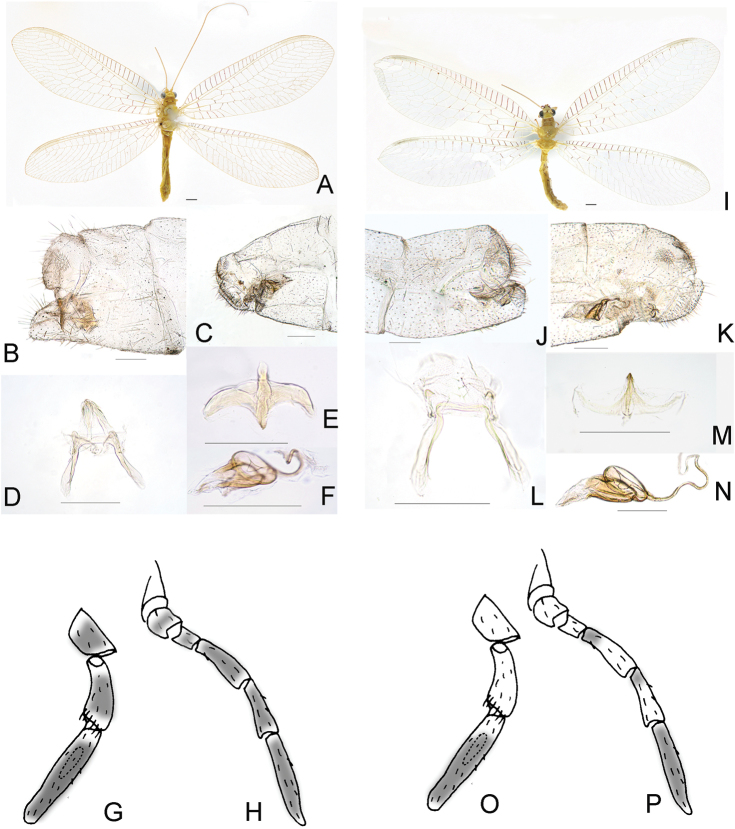
Photographs of habitus and genitalia of *Pseudomalladaprasinus* (Burmeister, 1839). Type **A** (**A–H**); type **B** (**I–P**); photographs of habitus (**A, I**); apex of abdomen in male (**B, J**); apex of abdomen in female (**C, K**); the complex of gonocoxites, gonapophyses and gonostyli 9, dorsal view (**D, L**); gonocoxites 10, dorsal view (**E, M**); spermatheca, lateral view (**F, N**); labial palps (**G, O**); maxillary palps (**H, P**). Scale bar: 1mm (**A, I**); 0.25 mm (**B–F, J–N**).

Among the green lacewing species herein studied, the bPTP_ML and bPTP_BS analyse resulted in 21 and 20 MOTUs, respectively (Figure 5B). Notably, bPTP_ML divided *P.cognatellus* (Okamoto, 1914) into two MOTUs (n=3) while the intraspecific distance is 0. Both solutions of the bPTP divided *Chrysopapallens* (Rambur, 1838) into two MOTUs (n=12), but the intraspecific divergence is relatively lower (1.1%). Furthermore, the bPTP species delimitation sorted *P.prasinus* into two MOTUs (i.e., types A and B). Meanwhile, *P.prasinus* of high intraspecific divergence (2.7%) was detected using K2P distance analysis (Suppl. material 8: Table S2). We carefully differentiated the morphological characters between these two types, and we found difference of color patterns on every segment of maxillary and labial palps. Those palps in type A are almost entirely black except for joints that are yellow, but in type B they are largely yellow except for the terminal segments and several joints that are black. Besides, the number of blackish markings on pronotum is different between types A and B. Type A has only one pair of blackish markings on the middle of pronotum, while type B possesses three pairs of additional blackish markings on the lateral margins of pronotum beside the medial pair of markings. Moreover, the apex of male sternum 9 in type A is narrowed distad, while in type B it is broader and subquadrate in lateral view. Nevertheless, no morphological difference was detected concerning the shape of the complex of gonocoxites, gonapophyses, and gonostyli 9 as well as the gonocoxites 10 (Figure 6). Thus, cryptic species may exist in *P.prasinus*, as mentioned in Duelli et al. (2017). The ABGD analysis resulted in 17 MOTUs, within which two species (i.e., *Pseudomallada* sp. 2 and sp. 3) were assigned into a same species.

### Hemerobiidae

The study resulted in 16 species of Hemerobiidae from Beijing although two species of them are undetermined. Eight species are newly recorded from Beijing, i.e., *Drepanepteryxalgida* (Erichson, 1851), *Hemerobiusbispinus* Banks, 1940, *Hemerobiusexoterus* Navás, 1936, *Hemerobiushumulinus* Linnaeus, 1758, *Hemerobiusjaponicus* Nakahara, 1915, *Hemerobiusmarginatus* (Stephens, 1836), *Hemerobiussubtriangulus* Yang, 1987 and *Sympherobiusmanchuricus* Nakahara, 1960. Seven species of them, except *D.algida*, were recorded from Beijing in an unpublished doctoral thesis (Zhao 2016) but not listed with their distribution from Beijing in Yang et al. (2018). *Drepanepteryxalgida* is also first recorded from China.

*Hemerobiushumulinus* and *Hemerobiusjaponicus* possess a minimum mean interspecific distance 5.3% (Suppl. material 9: Table S3). Meanwhile, *Hemerobiusjaponicus* and *Neuronema* sp. 1 demonstrate a maximum mean interspecific distance 21.8%. Results of species delimitation based on ABGD and bPTP_ML are consistent with our identification based on morphology. But in bPTP_BS analysis, all samples were sorted into 17 MOTUs, because *Neuronema* sp. 1 was divided into two MOTUs (n = 3; Figure 5C) even without any intraspecific divergence.

### Mantispidae and Dilaridae

The study obtained COI barcodes from two species of Mantispidae, i.e., *Eumantispaharmandi* (Navás, 1909) and *Mantispastyriaca* (Poda, 1761), and from one species of Dilaridae (*Dilarhastatus* Zhang, Liu, H. Aspöck & U. Aspöck, 2014; Wang et al. 2017).

### Myrmeleontidae and Ascalaphidae

Four species of Myrmeleontidae, i.e., *Dendroleonpantherinus* (Fabricius, 1787), *Distoleonnigricans* (Matsumura, 1905), *Euroleoncoreanus* (Okamoto, 1926) and *Myrmeleonbore* (Tjeder, 1941) and one species of Ascalaphidae [*Protidricerusjaponicus* (McLachlan, 1891)] from Beijing were studied. The consequence of two species delimitation methods is consistent with our identification based on morphology.

## Discussion

Within the past few years, DNA sequence-based approaches have become more and more popular for the assessment of biodiversity and identification of species, in particular where the traditional morphology-based identification is hard to apply (Taberlet et al. 2012). However, COI gene is known to be affected by several biases and is considered to better utilized in combination with, at least, other independent genes, but also with morphological, geographical or ecological data to clearly delimit species (Will et al. 2005; Ahrens et al. 2007; Padial et al. 2010; Hajibabaei et al. 2011).

The present DNA barcode library of Neuroptera from Beijing stands an important step not only for the molecular identification of lacewing species from Beijing but also for the future construction of DNA barcode database of Neuroptera from China. In light of obvious gap between intraspecific and interspecific genetic distance, the present COI barcode data allow unambiguous identification of almost all lacewing species from Beijing herein examined. Nevertheless, it should be noted that some other methods we tested for species delimitation (i.e., ABGD and bPTP) based on present barcode data may result in some problematic identification (see above results on *Semidalisaleyrodiformis*, *Pseudomallada* spp., and *Neuronema* sp. 1)

According to the updated catalogue of Neuroptera from China (Yang et al. 2018), 7 families, 12 subfamilies, 32 genera, and 47 species were recorded from Beijing. Here, Neuroptera from Beijing are composed of 7 families, 13 subfamilies, 37 genera, and 67 species (Suppl. material 6: File S2, excluding unidentified species).

Beijing is located at the eastern Palaearctic region. Among the 67 lacewing species from Beijing, 30 species (44.8% of total species) are distributed only from the Palaearctic region, while the remaining 37 species (55.2% of total species) occur in both Palaearctic and Oriental regions. The species of Chrysopidae and Hemerobiidae account for a great proportion (38.2% and 34.0% respectively) of Neuroptera in this study. They also represent substantial species numbers based on the checklist of Neuroptera from Beijing (28.4% and 25.4% respectively). Due to lack of specimens, species of Aleuropteryginae and many tribes of Myrmeleontidae were not studied here, but will be supplemented in our dataset in near future.

## Conclusions

Our study provided the first DNA barcode library of Neuroptera from Beijing, including 49 species (73% of all lacewing species recorded in Beijing). It is clearly indicated that the use of DNA barcodes for the identification of lacewing species is promising. The present dataset will be the first step toward the DNA barcoding of Chinese Neuroptera. It is also useful for the identification of immature stages and/or females of the lacewing species from Beijing. In future study, the DNA barcoding could be applied for comparison and assessment of lacewing species diversity and its dynamic change among different types of ecosystems and regions in Beijing for understanding the effect of urbanization on this important insect group.

## Supplementary materials

Supplementary material 1 Figure S1. Photographs of male genitalia of species of Coniopterygidae newly recorded from Beijing Data type: multimedia Explanation note: A. *Conwentziasinica* Yang, 1974; B. *Semidalisbicornis* Liu & Yang, 1993. Scale bar 0.5 mm. This is an open access article distributed under the terms of the Creative Commons Attribution License (CC BY 4.0), which permits unrestricted use, distribution, and reproduction in any medium, provided the original author and source are credited.Pan Yi, Pei Yu, Jingyi Liu, Huan Xu, Xingyue Liu

Supplementary material 2 Figure S2. Photographs of male genitalia of species of Chrysopidae newly recorded from Beijing Data type: multimedia Explanation note: A. *Nothochrysasinica* Yang, 1986; B. *Chrysopaintima* McLachlan, 1893; C. *Chrysoperlafurcifera* (Okamoto, 1914); D. *Chrysopidiaciliata* (Wesmael, 1841); E. *Pseudomalladacognatellus* (Okamoto, 1914); F. *Pseudomalladaqinlingensis* (Yang & Yang, 1989); G. *Ninetagrandis* Navás, 1915. Scale bar: 0.5 mm. This is an open access article distributed under the terms of the Creative Commons Attribution License (CC BY 4.0), which permits unrestricted use, distribution, and reproduction in any medium, provided the original author and source are credited.Pan Yi, Pei Yu, Jingyi Liu, Huan Xu, Xingyue Liu

Supplementary material 3 Figure S3. Photographs of male genitalia of species of Hemerobiidae newly recorded from Beijing Data type: multimedia Explanation note: A. *Drepanepteryxalgida* (Erichson, 1851); B. *Hemerobiusbispinus* Banks, 1940; C. *Hemerobiusexoterus* Navás, 1936; D. *Hemerobiushumulinus* Linnaeus, 1758; E. *Hemerobiusjaponicus* Nakahara, 1915; F. *Hemerobiusmarginatus* (Stephens, 1836); G. *Hemerobiussubtriangulus* Yang, 1987; H. *Sympherobiusmanchuricus* Nakahara, 1960. Scale bar: 0.5 mm. This is an open access article distributed under the terms of the Creative Commons Attribution License (CC BY 4.0), which permits unrestricted use, distribution, and reproduction in any medium, provided the original author and source are credited.Pan Yi, Pei Yu, Jingyi Liu, Huan Xu, Xingyue Liu

Supplementary material 4 Figure S4. Neighbor-joining tree based on the COI sequence dataset of Chrysopidae Data type: phylogenetic tree Explanation note: Neighbor-joining tree based on the COI sequence dataset of Chrysopidae. Only bootstrap supports (1,000 replicates) > 0.95 are labelled. This is an open access article distributed under the terms of the Creative Commons Attribution License (CC BY 4.0), which permits unrestricted use, distribution, and reproduction in any medium, provided the original author and source are credited.Pan Yi, Pei Yu, Jingyi Liu, Huan Xu, Xingyue Liu

Supplementary material 5 File S1. List of all specimens used in this study, including GenBank accession numbers Data type: molecular data This is an open access article distributed under the terms of the Creative Commons Attribution License (CC BY 4.0), which permits unrestricted use, distribution, and reproduction in any medium, provided the original author and source are credited.Pan Yi, Pei Yu, Jingyi Liu, Huan Xu, Xingyue Liu

Supplementary material 6 File S2. Checklist of the species of Neuroptera from Beijing Data type: species data This is an open access article distributed under the terms of the Creative Commons Attribution License (CC BY 4.0), which permits unrestricted use, distribution, and reproduction in any medium, provided the original author and source are credited.Pan Yi, Pei Yu, Jingyi Liu, Huan Xu, Xingyue Liu

Supplementary material 7 Table S1. Intraspecific and interspecific divergence of Coniopterygidae based on COI barcode sequences (%) Data type: molecular data Explanation note: The range of interspecific distance = means interspecific distance ± standard error. N/A indicates intraspecific distance not available because only one specimen was sequenced. This is an open access article distributed under the terms of the Creative Commons Attribution License (CC BY 4.0), which permits unrestricted use, distribution, and reproduction in any medium, provided the original author and source are credited.Pan Yi, Pei Yu, Jingyi Liu, Huan Xu, Xingyue Liu

Supplementary material 8 Table S2. Intraspecific and interspecific divergence of Chrysopidae based on COI barcode sequences (%) Data type: molecualr data Explanation note: The range of interspecific distance = mean interspecific distance ± standard error. N/A indicates intraspecific distance not available because only one specimen was sequenced. This is an open access article distributed under the terms of the Creative Commons Attribution License (CC BY 4.0), which permits unrestricted use, distribution, and reproduction in any medium, provided the original author and source are credited.Pan Yi, Pei Yu, Jingyi Liu, Huan Xu, Xingyue Liu

Supplementary material 9 Table S3. Intraspecific and interspecific divergence of Hemerobiidae based on COI barcode sequences (%) Data type: molecular data Explanation note: Tthe range of interspecific distance = mean interspecific distance ± standard error. N/A indicates intraspecific distance not available because only one specimen was sequenced. This is an open access article distributed under the terms of the Creative Commons Attribution License (CC BY 4.0), which permits unrestricted use, distribution, and reproduction in any medium, provided the original author and source are credited.Pan Yi, Pei Yu, Jingyi Liu, Huan Xu, Xingyue Liu

Supplementary material 10 Table S4. Intraspecific and interspecific divergence of Myrmeleontidae and Ascalaphidae based on COI barcode sequences (%) Data type: molecular data Explanation note: The range of interspecific distance = mean interspecific distance ± standard error. N/A indicates intraspecific distance not available because only one specimen was sequenced. This is an open access article distributed under the terms of the Creative Commons Attribution License (CC BY 4.0), which permits unrestricted use, distribution, and reproduction in any medium, provided the original author and source are credited.Pan Yi, Pei Yu, Jingyi Liu, Huan Xu, Xingyue Liu

## References

[B1] Abdrabou S (2008) Evaluation of the green lacewing, *Chrysoperlacarnea* (Stephens) (Neuroptera: Chrysopidae) against aphids on different crops. Journal of Biological Control 22: 299–310.

[B2] Ahrens D Monaghan MT Vogler AP (2007) DNA-based taxonomy for associating adults and larvae in multi-species assemblages of chafers (Coleoptera: Scarabaeidae). Molecular Phylogenetics and Evolution 44: 436–449. 10.1016/j.ympev.2007.02.024 17420144

[B3] Aspöck H Aspöck U Hölzel H (1980) Die Neuropteren Europas. Goecke & Evers, Krefeld, 495 pp [vol. 1], 355 pp [vol. 2].

[B4] Aspöck U Haring E Aspöck H (2012) The phylogeny of the Neuropterida: long lasting and current controversies and challenges (Insecta: Endopterygota). Arthropod Systematics & Phylogeny 70: 119–129.

[B5] Badano D Acevedo F Pantaleoni RA Monserrat VJ (2016) *Myrmeleonalmohadarum* sp. nov. from Spain and North Africa, with description of the larva (NeuropteraMyrmeleontidae). Zootaxa 4196: 210–220. 10.11646/zootaxa.4196.2.2 27988672

[B6] Bezerra GCD Santacecília LVC Carvalho CF Souza B (2006) Biological aspects of the adult stage of *Chrysoperlaexterna* (Hagen, 1861) (Neuroptera: Chrysopidae) originating from the larvae fed *Planococcuscitri* (Risso, 1813) (Hemiptera: Pseudococcidae). Ciência Agrotecnologia 30: 603–610. 10.1590/S1413-70542006000400002

[B7] Burns JM Janzen DH Hajibabaei M Hallwachs W Hebert PDN (2008) DNA barcodes and cryptic species of skipper butterflies in the genus *Perichares* in Area de Conservacion Guanacaste, Costa Rica. Proceedings of the National Academy of Sciences of the United States of America 105: 6350–6355. 10.1073/pnas.0712181105 18436645 PMC2359806

[B8] Choi MY Mochizuki A Henry CS (2015) The green lacewing, *Chrysoperlanipponensis* in nature and in an insectary population in Korea: Song types and mitochondrial COI haplotypes. Journal of Asia-Pacific Entomology 18: 151–155. 10.1016/j.aspen.2014.12.009

[B9] Dai YT Winterton SL Garzón-Orduña IJ Liang FY Liu XY (2017) Mitochondrial phylogenomic analysis resolves the subfamily placement of enigmatic green lacewing genus *Nothancyla* (Neuroptera: Chrysopidae). Austral Entomology 56: 322–331. 10.1111/aen.12220

[B10] Duelli P Henry CS Hayashi M Nomura M Mochizuki A (2017) Molecular phylogeny and morphology of *Pseudomallada* (Neuroptera: Chrysopidae), one of the largest genera within Chrysopidae. Zoological Journal of the Linnean Society 180: 556–569. 10.1093/zoolinnean/zlw008

[B11] Duelli P Johnson JB Waldburger M Henry CS (2016) A New Look at Adaptive Body Coloration and Color Change in “Common Green Lacewings” of the Genus *Chrysoperla* (Neuroptera: Chrysopidae). Annals of the Entomological Society of America 107: 382–388. 10.1603/AN13139

[B12] Engel MS Winterton SL Breitkreuz L (2018) Phylogeny and Evolution of Neuropterida: Where Have Wings of Lace Taken Us? Annual Review of Entomology 63: 531–551. 10.1146/annurev-ento-020117-043127 29324039

[B13] Folmer O Black M Hoeh W Lutz R Vrijenhoek R (1994) DNA primers for amplification of mitochondrial cytochrome c oxidase subunit I from diverse metazoan invertebrates. Molecular Marine Biology and Biotechnology 3: 294–299. 7881515

[B14] Goolsby JA Rose M Morrison RK Woolley JB (2000) Augmentative biological control of longtailed mealybug by *Chrysoperlarufilabris* (Burmeister) in the interior plantscape. Southwest Entomologist 25: 15–19.

[B15] Hajibabaei M Shokralla S Zhou X Singer GAC Baird DJ (2011) Environmental barcoding: a next-generation sequencing approach for biomonitoring applications using river benthos. Plos One 6: e17497. 10.1371/journal.pone.0017497 PMC3076369 21533287

[B16] Hebert PDN Cywinska A Ball SL Dewaard JR (2003a) Biological identifications through DNA barcodes. Proceedings of the Royal Society B: Biological Sciences 270: 313–321. https:/10.1098/rspb.2002.2218 PMC1691236 12614582

[B17] Hebert PDN Ratnasingham S Dewaard JR (2003b) Barcoding animal life: cytochrome c oxidase subunit 1 divergences among closely related species. Proceedings of the Royal Society of London Series B: Biological Sciences 270: S96–S99. 10.1098/rsbl.2003.0025 PMC1698023 12952648

[B18] Henry CS Brooks SJ Duelli P Johnson JB Wells MLM Mochizuki A (2013) Obligatory duetting behaviour in the *Chrysoperlacarnea*-group of cryptic species (Neuroptera: Chrysopidae): its role in shaping evolutionary history. Biological Reviews 88: 787–808. 10.1111/brv.12027 23433087

[B19] Henry CS Brooks SJ Johnson JB Mochizuki A Duelli P (2014) A new cryptic species of the *Chrysoperlacarnea* group (Neuroptera: Chrysopidae) from western Asia: parallel speciation without ecological adaptation. Systematic Entomology 39: 380–393. 10.1111/syen.12061

[B20] Henry CS Wells MM (2010) Acoustic niche partitioning in two cryptic sibling species of *Chrysoperla* green lacewings that must duet before mating. Animal Behaviour 80: 991–1003. 10.1016/j.anbehav.2010.08.021

[B21] Huemer P Karsholt O Mutanen M (2014) DNA barcoding as a screening tool for cryptic diversity: an example from *Caryocolum*, with description of a new species (Lepidoptera, Gelechiidae). Zookeys 404: 91–111. 10.3897/zookeys.404.7234 PMC4023261 24843272

[B22] Kimura M (1980) A simple method for estimating evolutionary rates of base substitutions through comparative studies of nucleotide sequences. Journal of Molecular Evolution 16: 111–120. 10.1007/BF01731581 7463489

[B23] Liu ZQ (2003) Studies on the Taxonomy and Taxonomic information system of Coniopterygidae from China. PhD thesis, Beijing, China: China Agricultural University.

[B24] McEwen PK New TR Whittington AE (2001) Lacewings in the crop environment. Cambridge University Press, Cambridge, 546 pp.

[B25] Messelink GJ Vijverberg R Leman A Janssen A (2016) Biological control of mealybugs with lacewing larvae is affected by the presence and type of supplemental prey. Biocontrol 61: 555–565. 10.1007/s10526-016-9739-y

[B26] Misof B Liu S Meusemann K Peters RS Donath A Mayer C et al. (2014) Phylogenomics resolves the timing and pattern of insect evolution. Science 346: 763–767. 10.1126/science.1257570 25378627

[B27] Morales AC Freitas S (2010) Haplotype characterization of the COI mitochondrial gene in *Chrysoperlaexterna* (Neuroptera: Chrysopidae) from different environments in Jaboticabal, state of São Paulo, Southeastern Brazil. Brazilian Journal of Biology 70: 1115–1121. 10.1590/S1519-69842010000500030 21180923

[B28] Morinière J Hendrich L Hausmann A Hebert P Haszprunar G Gruppe A (2014) Barcoding Fauna Bavarica: 78% of the Neuropterida Fauna Barcoded!. Plos One 9: e109719. 10.1371/journal.pone.0109719 PMC4186837 25286434

[B29] Oswald JD (chief editor) (2018) Lacewing Digital Library: Neuropterida Species of the World. http://lacewing.tamu.edu/SpeciesCatalog/Main [Accessed on 8 October 2018]

[B30] Padial JM Miralles A De la Riva I Vences M (2010) The integrative future of taxonomy. Frontiers in Zoology 7: 1–14. 10.1186/1742-9994-7-16 20500846 PMC2890416

[B31] Pantaleoni RA Badano D (2012) *Myrmeleonpunicanus* n. sp. a new pit-building antlion (NeuropteraMyrmeleontidae) from Sicily and Pantelleria. Bulletin of Insectology 65: 139–148.

[B32] Price BW Henry CS Hall AC Mochizuki A Duelli P Brooks SJ (2015) Singing from the Grave: DNA from a 180 Year Old type Specimen Confirms the Identity of *Chrysoperlacarnea* (Stephens). Plos One 10: e0121127. 10.1371/journal.pone.0121127 PMC4390323 25853856

[B33] Puillandre N Lambert A Brouillet S Achaz G (2012) ABGD, Automatic Barcode Gap Discovery for primary species delimitation. Molecular Ecology 21: 1864–1877. 10.1111/j.1365-294X.2011.05239.x 21883587

[B34] Saitou N Nei M (1987) The neighbor-joining method: a new method for reconstructing phylogenetic trees. Molecular Biology and Evolution 4: 406–425. 3447015 10.1093/oxfordjournals.molbev.a040454

[B35] Sato T Takada H (2004) Biological studies on three *Micromus* species in Japan (Neuroptera: Hemerobiidae) to evaluate their potential as biological control agents against aphids: 1. thermal effects on development and reproduction. Applied Entomology and Zoology 39: 417–425. 10.1303/acz.2004.417

[B36] Smith MA Woodley NE Janzen DH Hallwachs W Hebert PDN (2006) DNA barcodes reveal cryptic host-specificity within the presumed polyphagous members of a genus of parasitoid flies (Diptera: Tachinidae). Proceedings of the National Academy of Sciences 103: 3657–3662. 10.1073/pnas.0511318103 PMC1383497 16505365

[B37] Song C Lin XL Wang Q Wang XH (2018) DNA barcodes successfully delimit morphospecies in a superdiverse insect genus. Zoologica Scripta 47: 311–324. 10.1111/zsc.12284

[B38] Taberlet P Coissac E Pompanon F Brochmann C Willerslev E (2012) Towards next-generation biodiversity assessment using DNA metabarcoding. Molecular Ecology 21: 2045–2050. 10.1111/j.1365-294X.2012.05470.x 22486824

[B39] Tamura K Peterson D Peterson N Stecher G Nei M Kumar S (2011) MEGA5: molecular evolutionary genetics analysis using maximum likelihood, evolutionary distance, and maximum parsimony methods. Molecular Biology and Evolution 28: 2731–2739. 10.1093/molbev/msr121 21546353 PMC3203626

[B40] Thompson JD Higgins DG Gibson TJ (1994) CLUSTAL W: improving the sensitivity of progressive multiple sequence alignment through sequence weighting, position-specific gap penalties and weight matrix choice. Nucleic Acids Research 22: 4673–4680. 10.1093/nar/22.22.4673 7984417 PMC308517

[B41] Vidya M Lingappa S Patil RK Ramegowda GK (2010) Biology and feeding potential of *Micromustimidus* Hagen (Neuroptera: Hemerobiidae) on sugarcane woolly aphid, Ceratovacuna lanigera Zehntner. Karnataka Journal of Agricultural Sciences 23: 246–248.

[B42] Wang XL Zhan QB Wang AQ (2018) Fauna Sinica, Insecta, Vol. 68, Neuroptera, Myrmeleontoidea. Science Press, Beijing, 323 pp.

[B43] Wang YY Liu XY Winterton SL Yan Y Aspöck U Aspöck H (2017) Mitochondrial phylogenomics illuminates the evolutionary history of Neuropterida. Cladistics 33: 617–636. 10.1111/cla.12186 34724753

[B44] Will KW Mishler BD Wheeler QD (2005) The perils of DNA barcoding and the need for integrative taxonomy. Systematic Biology 54: 844–851. 10.1080/10635150500354878 16243769

[B45] Winterton SL Lemmon AR Gillung JP Garzon IJ Badano D Bakkes DK et al. (2018) Evolution of lacewings and allied orders using anchored phylogenomics (Neuroptera, Megaloptera, Raphidioptera). Systematic Entomology 43: 330–354. 10.1111/syen.12278

[B46] Yang D Liu XY Yang XK et al. (2018) Catalogue of Superorder Neuropterida (Insecta) from China. Science Press, Beijing, 172 pp.

[B47] Yang XK Yang JK Li WZ (2005) Fauna Sinica, Insecta vol. 39, Neuroptera, Chrysopidae. Science Press, Beijing, 420 pp.

[B48] Zhang W Liu XY Aspöck H Aspöck U (2014) Revision of Chinese Dilaridae (Insecta: Neuroptera) (Part I): species of the genus *Dilar* Rambur from northern China. Zootaxa 3753: 10–24. 10.11646/zootaxa.3753.1.2 24872276

[B49] Zhang J Kapli P Pavlidis P Stamatakis A (2013) A general species delimitation method with applications to phylogenetic placements. Bioinformatics 29: 2869–2876. 10.1093/bioinformatics/btt499 23990417 PMC3810850

[B50] Zhao Y (2016) Systematics of Family Hemerobiidae from China (Intesta: Neuroptera, Hemerobiidae). PhD Thesis, Beijing, China: China Agricultural University.

[B51] Zhao Y Yan B Liu Z (2013) New species of *Neuronema* Mclachlan, 1869 from China (Neuroptera, Hemerobiidae). Zootaxa 3710: 557–564. 10.11646/zootaxa.3710.6.2 26106710

