# Supplementary figures and images for: A DNA barcode reference library of Neuroptera (Insecta, Neuropterida) from Beijing
Source: Zookeys. 2018 Dec 17;(807):127–47. doi: 10.3897/zookeys.807.29430 (PMC6305355; doi:10.3897/zookeys.807.29430)

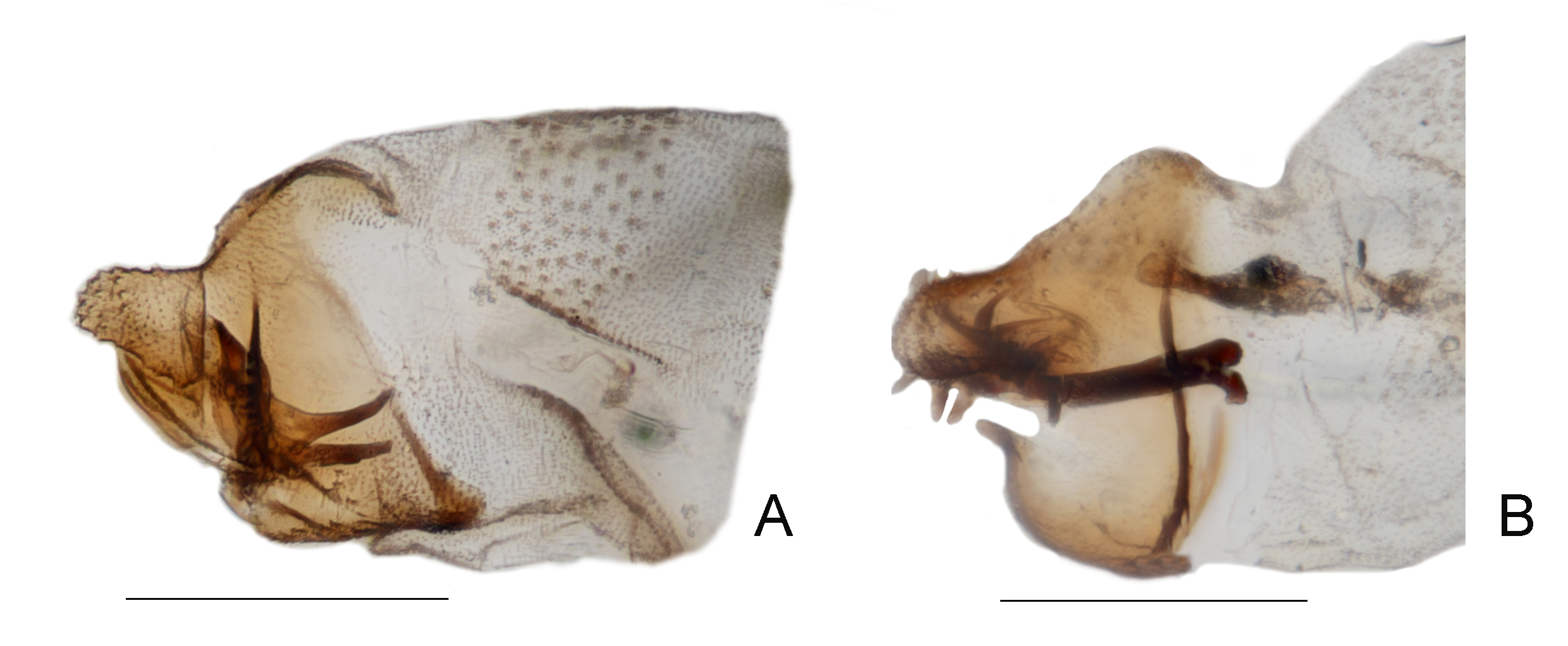

Supplement: Supplementary material 1 — Figure S1. Photographs of male genitalia of species of Coniopterygidae newly recorded from Beijing [file zookeys-807-127-s001.png]

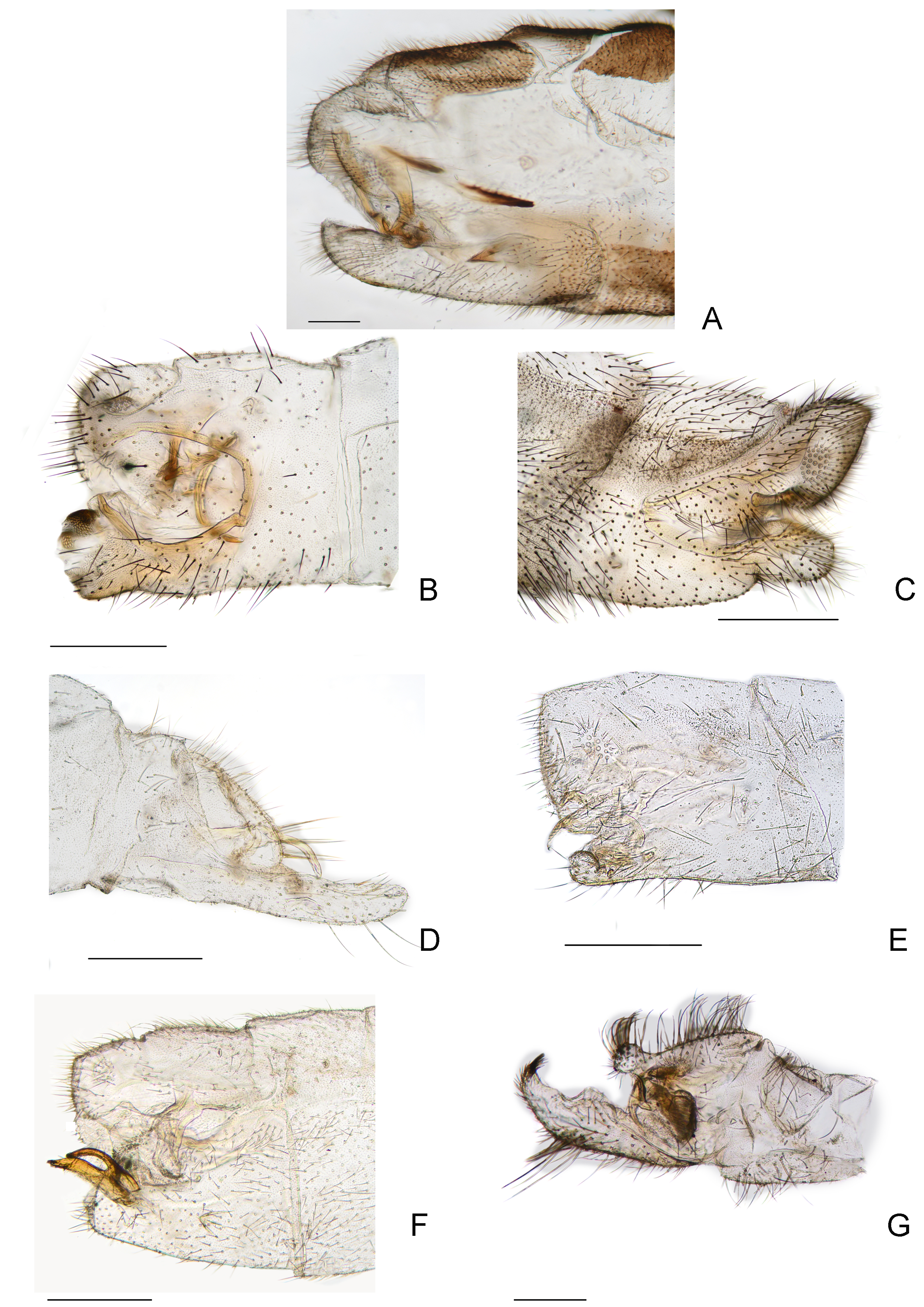

Supplement: Supplementary material 2 — Figure S2. Photographs of male genitalia of species of Chrysopidae newly recorded from Beijing [file zookeys-807-127-s002.png]

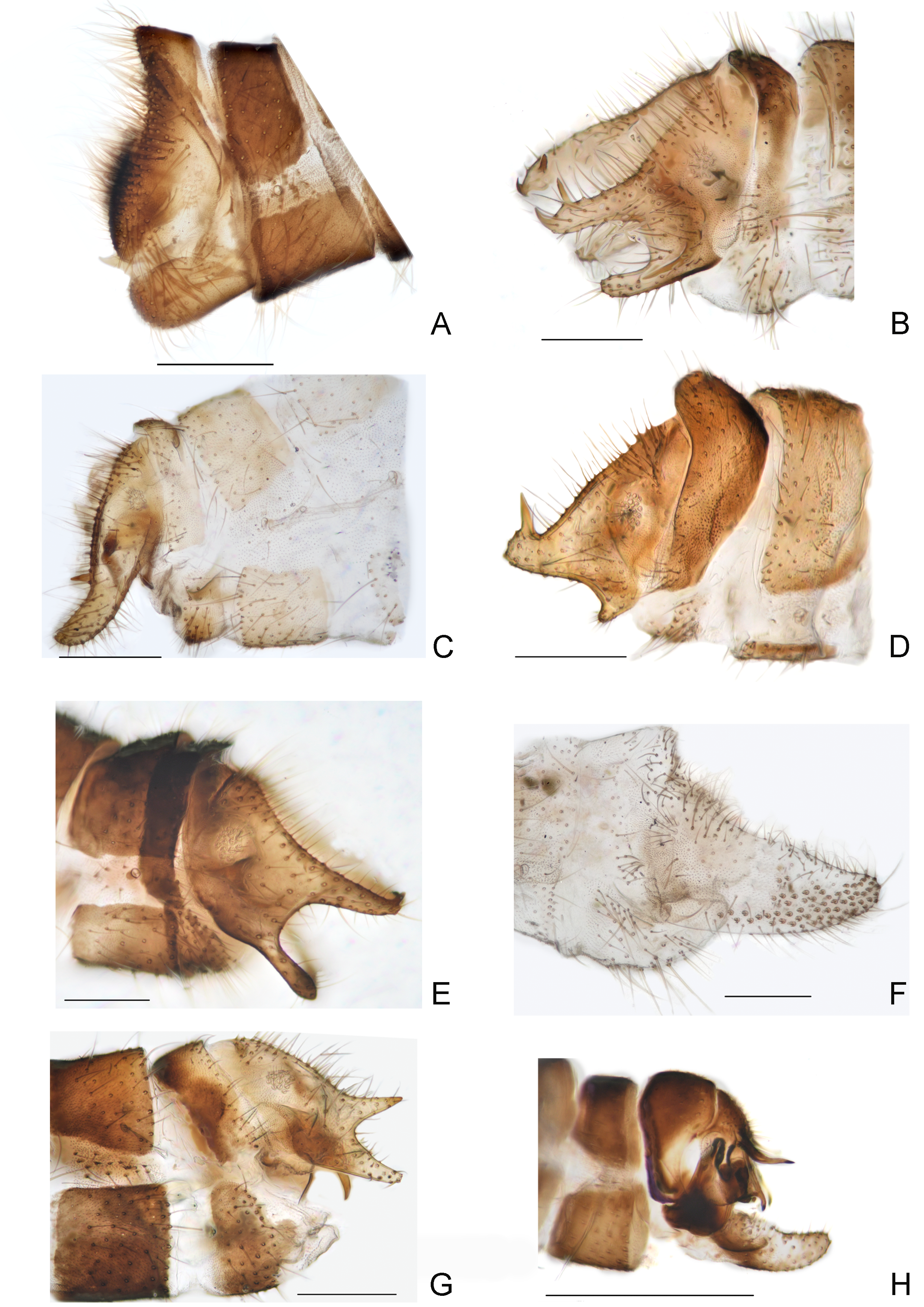

Supplement: Supplementary material 3 — Figure S3. Photographs of male genitalia of species of Hemerobiidae newly recorded from Beijing [file zookeys-807-127-s003.png]

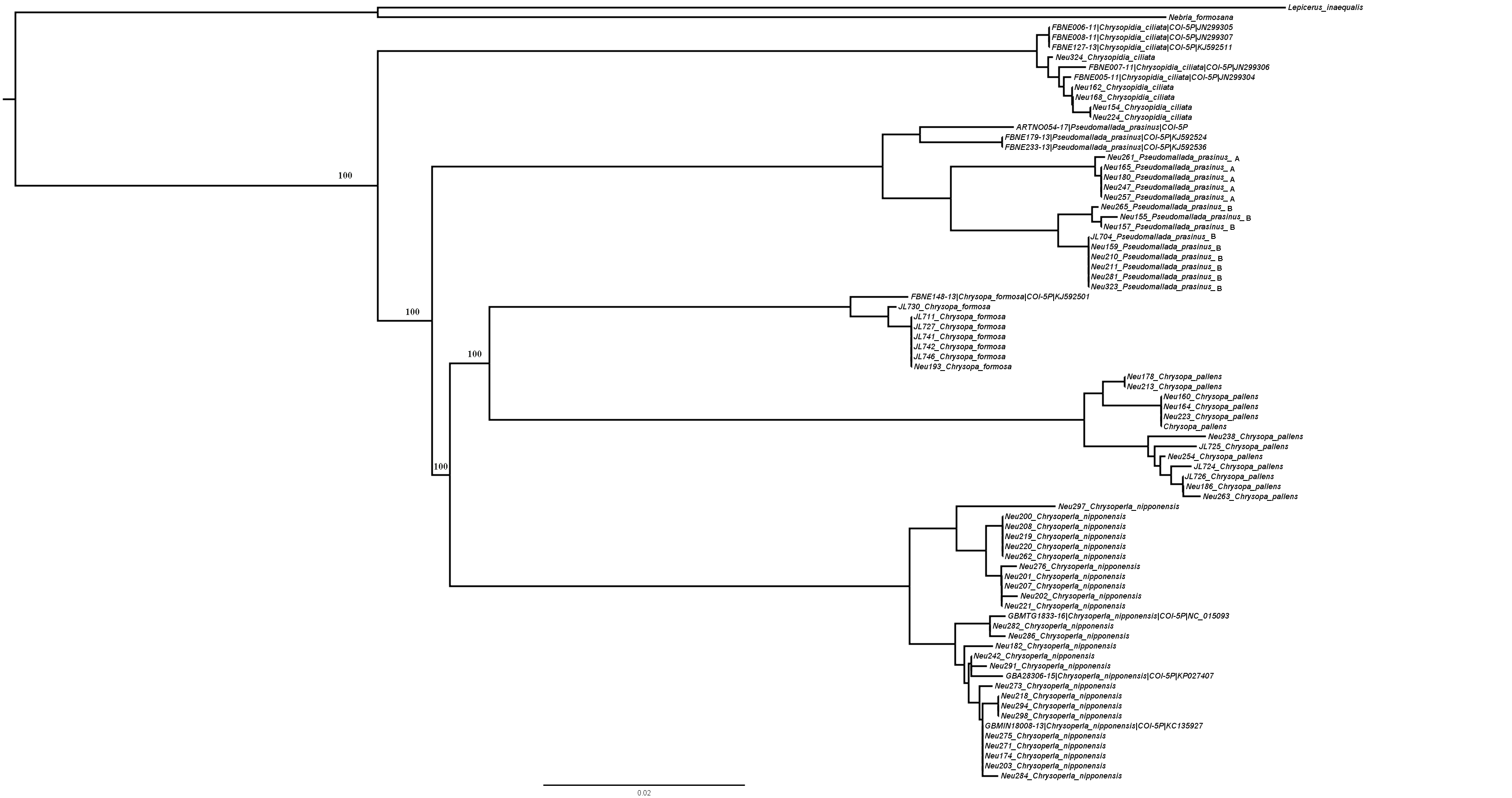

Supplement: Supplementary material 4 — Figure S4. Neighbor-joining tree based on the COI sequence dataset of Chrysopidae [file zookeys-807-127-s004.png]
