# Supplementary material for: A DNA barcode reference library of Neuroptera (Insecta, Neuropterida) from Beijing
Source: Zookeys. 2018 Dec 17;(807):127–47. doi: 10.3897/zookeys.807.29430 (PMC6305355; doi:10.3897/zookeys.807.29430)
Supplement: Supplementary material 5 — File S1. List of all specimens used in this study, including GenBank accession numbers [file zookeys-807-127-s005.doc]

|  | | | | | |
| --- | --- | --- | --- | --- | --- |
| Species | 1. *P. japonicus* | 2. *D. pantherinus* | 3. *D. nigricans* | 4. *E. coreanus* | 5. *M. bore* |
| 1 | 0. 1 | 15.9–19.5 | 15.6–19.1 | 16.5–20.0 | 16.5–20.2 |
| 2 |  | 0.8 | 16.8–20.5 | 15.2–18.6 | 15.5–19.0 |
| 3 |  |  | N/A | 13.3–16.3 | 15.3–18.6 |
| 4 |  |  |  | 0.2 | 11.8–14.6 |
| 5 |  |  |  |  | N/A |
