# Supplementary material for: A DNA barcode reference library of Neuroptera (Insecta, Neuropterida) from Beijing
Source: Zookeys. 2018 Dec 17;(807):127–47. doi: 10.3897/zookeys.807.29430 (PMC6305355; doi:10.3897/zookeys.807.29430)
Supplement: Supplementary material 7 — Table S1. Intraspecific and interspecific divergence of Coniopterygidae based on COI barcode sequences (%) [file zookeys-807-127-s007.doc]

|  | | | | | | | |
| --- | --- | --- | --- | --- | --- | --- | --- |
| Species | 1. *C. sinica* | 2. *S. aleyrodiformis* | 3. *S. bicornis* | 4. *C. plagiotropa* | 5. *C. pygmaea* | 6*. Coniopteryx* sp. 1 | 7. *Coniopteryx* sp. 2 |
| 1 | 0 | 18.8–22.6 | 17.3–21.2 | 16.5–20.1 | 17.8–21.7 | 18.9–22.9 | 16.8–19.8 |
| 2 |  | 0 | 14.8–18.1 | 21.2–25.3 | 17.7–21.4 | 19.4–23.5 | 21.0–25.2 |
| 3 |  |  | N/A | 18.1–22.0 | 17.4–21.2 | 19.4–23.5 | 17.7–21.7 |
| 4 |  |  |  | 0.1 | 16.3–19.9 | 16.0–19.5 | 14.9–18.3 |
| 5 |  |  |  |  | 0.5 | 15.8–19.4 | 16.7–20.4 |
| 6 |  |  |  |  |  | N/A | 9.5–12.3 |
| 7 |  |  |  |  |  |  | N/A |
