# Supplementary material for: A DNA barcode reference library of Neuroptera (Insecta, Neuropterida) from Beijing
Source: Zookeys. 2018 Dec 17;(807):127–47. doi: 10.3897/zookeys.807.29430 (PMC6305355; doi:10.3897/zookeys.807.29430)
Supplement: Supplementary material 8 — Table S2. Intraspecific and interspecific divergence of Chrysopidae based on COI barcode sequences (%) [file zookeys-807-127-s008.doc]

| 18 | 17 | 16 | 15 | 14 | 13 | 12 | 11 | 10 | 9 | 8 | 7 | 6 | 5 | 4 | 3 | 2 | 1 | Species |
| --- | --- | --- | --- | --- | --- | --- | --- | --- | --- | --- | --- | --- | --- | --- | --- | --- | --- | --- |
|  |  |  |  |  |  |  |  |  |  |  |  |  |  |  |  |  | 0.01 | 1*.C. formosa* |
|  |  |  |  |  |  |  |  |  |  |  |  |  |  |  |  | 0.5 | 9.2–11.7 | 2.*C. intima* |
|  |  |  |  |  |  |  |  |  |  |  |  |  |  |  | 1.2 | 9.5–12.2 | 9.9–12.5 | 3.*C. pallens* |
|  |  |  |  |  |  |  |  |  |  |  |  |  |  | 0.1 | 11.8–14.8 | 11.2–14.0 | 9.2–11.8 | 4.*C. furcifera* |
|  |  |  |  |  |  |  |  |  |  |  |  |  | 1.1 | 7.6–9.8 | 11.2–14.0 | 11.4–14.3 | 8.8–11.2 | 5.*C. nipponensis* |
|  |  |  |  |  |  |  |  |  |  |  |  | 0.2 | 11.8–14.7 | 12.8–15.9 | 12.3–15.3 | 14.4–17.7 | 10.3–13.0 | 6.*C. ciliata* |
|  |  |  |  |  |  |  |  |  |  |  | 0.1 | 11.4–14.1 | 10–12.6 | 10.7–13.5 | 11.0–13.8 | 12.0–15.0 | 12.1–15.1 | 7.*I. beijingana* |
|  |  |  |  |  |  |  |  |  |  | 0.6 | 9.0–11.4 | 10.1–12.9 | 9.8–12.4 | 8.9–11.4 | 11.3–14.1 | 12.2–15.3 | 10.1–12.9 | 8 *M. flavimaculus* |
|  |  |  |  |  |  |  |  |  | 0.2 | 10.4–13.0 | 10.4–13.1 | 11.9–14.9 | 9.6–12.2 | 9.3–11.9 | 11.0–13.8 | 11.9–14.9 | 9.9–12.4 | 9.*N. grandis* |
|  |  |  |  |  |  |  |  | 0.2 | 6.1–8.1 | 8.2–10.4 | 9.1–11.7 | 9.5–12.2 | 8.0–10.3 | 8.8–11.3 | 10.4–12.9 | 10.9–13.7 | 8.5–10.9 | 10.*N. shaanxiensis* |
|  |  |  |  |  |  |  | 0 | 9.8–12.5 | 10.8–13.5 | 9.8–12.5 | 11.1–13.9 | 12.2–15.3 | 9.7–12.3 | 9.5–12 | 10.6–13.3 | 11.7–14.7 | 10.5–13.1 | 11.*P. cognatellus* |
|  |  |  |  |  |  | 0.2 | 7.1–9.5 | 8.7–11.2 | 10.9–13.7 | 10.5–13.2 | 10.2–12.8 | 14.2–17.5 | 10.0–12.6 | 8.0–10.3 | 12.3–15.4 | 11.7–14.5 | 11.5–14.2 | 12.*P. illota* |
|  |  |  |  |  | 2.7 | 8.5–10.8 | 7.7–10.0 | 10.3–12.8 | 11.4–14.1 | 9.7–12.5 | 12.7–15.6 | 12.9–15.8 | 10.7–13.3 | 9.8–12.4 | 11.1–13.9 | 11.0–13.7 | 10.3–12.9 | 13.*P. prasinus* |
|  |  |  |  | 0 | 10.5–13.2 | 10.8–13.7 | 8.1–10.5 | 7.5–9.9 | 8.4–11.0 | 8.1–10.5 | 10.4–13.1 | 10.2–13.2 | 9.4–12.0 | 9.4–11.9 | 10.8–13.6 | 12.3–15.4 | 9.4–12.0 | 14.*P. qinlingensis* |
|  |  |  | N/A | 9.4–11.9 | 8.0–11.4 | 5.4–7.4 | 7.9–10.3 | 9.2–11.7 | 11.5–14.4 | 8.2–10.8 | 9.2–11.7 | 12.3–15.2 | 9.4–11.9 | 8.9–11.4 | 9.6–12.1 | 10.8–13.6 | 9.5–12.1 | 15.*Pseudomallada* sp. 1 |
|  |  | 0.2 | 10.8–13.5 | 11–13.9 | 11.0–13.8 | 9.9–12.7 | 9.2–11.9 | 9.0–11.5 | 10.6–13.4 | 9.4–12.1 | 11.3–14.1 | 11.4–14.3 | 10.4–13.0 | 11.4–14.2 | 12.6–15.6 | 13.2–16.3 | 11.3–14.1 | 16.*Pseudomallada* sp. 2 |
|  | 0.2 | 2.9–4.4 | 9.9–12.6 | 10.1–12.9 | 10.3–12.9 | 9.1–11.7 | 8.1–10.6 | 8.4–10.9 | 11.6–14.6 | 8.3–10.8 | 10.7–13.5 | 10.9–13.8 | 9.3–11.3 | 10.8–13.6 | 11.8–14.9 | 13.0–16.3 | 10.8–13.6 | 17.*Pseudomallada* sp. 3 |
| 0.1 | 8.8–11.5 | 9.6–12.2 | 9.6–12.4 | 10.0–12.8 | 9.1–11.7 | 9.7–12.5 | 8.1–10.6 | 9.3–11.9 | 11.3–14.3 | 8.5–11.1 | 10.1–12.9 | 11.3–14.2 | 9.6–12.2 | 8.5–11.0 | 10.8–13.6 | 11.7–14.8 | 9.9–12.6 | 18.*N. sinica* |
