# Supplementary material for: A DNA barcode reference library of Neuroptera (Insecta, Neuropterida) from Beijing
Source: Zookeys. 2018 Dec 17;(807):127–47. doi: 10.3897/zookeys.807.29430 (PMC6305355; doi:10.3897/zookeys.807.29430)
Supplement: Supplementary material 9 — Table S3. Intraspecific and interspecific divergence of Hemerobiidae based on COI barcode sequences (%) [file zookeys-807-127-s009.doc]

| 16 | 15 | 14 | 13 | 12 | 11 | 10 | 9 | 8 | | 7 | 6 | 5 | 4 | 3 | 2 | 1 | Species |
| --- | --- | --- | --- | --- | --- | --- | --- | --- | --- | --- | --- | --- | --- | --- | --- | --- | --- |
|  |  |  |  |  |  |  |  |  | |  |  |  |  |  |  | 0 | 1. *D. algida* |
|  |  |  |  |  |  |  |  |  | |  |  |  |  |  | N/A | 7.9–10.4 | 2. *D. phalaenoides* |
|  |  |  |  |  |  |  |  |  | |  |  |  |  | 0。08 | 16.7–20.4 | 13.3–16.7 | 3. *N. laminatum* |
|  |  |  |  |  |  |  |  |  | |  |  |  | 0 | 14.7–18.3 | 18.7–22.4 | 17.3–21.1 | 4. *Neuronema* sp. 1 |
|  |  |  |  |  |  |  |  |  | |  |  | 0.9 | 20.4–24.6 | 15.6–19.0 | 15.4–18.8 | 15.4–18.9 | 5. *H. bispinus* |
|  |  |  |  |  |  |  |  |  | |  | 0.7 | 11.2–14.1 | 18.7–22.7 | 15.8–19.3 | 18.2–21.8 | 16.6–20.3 | 6. *H. exoterus* |
|  |  |  |  |  |  |  |  |  | | 0.1 | 11.5–14.4 | 4.4–6.1 | 19.8–23.8 | 15.4–18.9 | 14.8–18.1 | 14.3–17.5 | 7. *H. humulinus* |
|  |  |  |  |  |  |  |  | N/A | | 4.5–6.3 | 11.1–13.9 | 4.9–6.7 | 20.7–24.8 | 15.8–19.3 | 13.8–17.0 | 13.8–17.0 | 8. *H. japonicus* |
|  |  |  |  |  |  |  | 0.5 | 10.4–13.2 | 9.2–11.8 | | 10.5–13.3 | 9.8–12.5 | 17.9–21.7 | 12.8–15.9 | 16.8–20.4 | 15.3–18.7 | 9. *H. marginatus* |
|  |  |  |  |  |  | 0.08 | 12.6–15.6 | 8.7–11.3 | | 8.7–11.2 | 12.2–15.2 | 9.2–11.8 | 20.5–24.6 | 15.8–19.3 | 14.9–18.2 | 16.2–19.7 | 10. *H. subtriangulus* |
|  |  |  |  |  | 0.5 | 9.7–12.6 | 11.9–14.8 | 9.1–11.6 | | 9.9–12.6 | 12.4–15.4 | 10.2–13.0 | 19.3–23.1 | 17.3–21.0 | 15.8–19.2 | 16.9–20.5 | 11. *Hemerobius* sp. 1 |
|  |  |  |  | 0.2 | 11.3–14.1 | 10.6–13.5 | 10.8–13.5 | 10.8–13.6 | | 10.5–13.3 | 11.1–13.9 | 11.5–14.4 | 17.6–21.2 | 16.1–19.6 | 13.1–16.2 | 12.9–16.0 | 12. *W. bihamitus* |
|  |  |  | 1.1 | 14.0–17.1 | 18.0–21.7 | 16.9–20.4 | 14.3–17.6 | 15.2–18.6 | | 15.6–19.1 | 18.0–21.7 | 15.2–18.6 | 19.3–23.1 | 15.9–19.4 | 15.5–19.0 | 11.7–14.6 | 13. *S. yunpinus* |
|  |  | 0.5 | 11.7–14.5 | 13.6–16.7 | 15.4–18.8 | 14.2–17.5 | 16.5–20.0 | 15.0–18.4 | | 14.0–17.2 | 18.8–22.5 | 14.8–18.0 | 20.7–24.6 | 16.8–20.3 | 13.9–17.1 | 11.8–14.7 | 14. *S. manchuricus* |
|  | 0.2 | 16.3–19.8 | 16.6–20.2 | 16.5–20.0 | 16.7–20.2 | 19.2–23.2 | 17.0–20.5 | 19.0–23.0 | | 18.7–22.4 | 17.7–21.4 | 18.3–22.0 | 18.7–22.6 | 16.1–19.4 | 19.7–23.4 | 17.3–20.9 | 15. *M. angulatus* |
| 0.06 | 16.5–20.2 | 17.8–21.6 | 15.9–19.4 | 16.2–19.7 | 19.6–23.5 | 18.9–22.8 | 16.5–20.4 | 19.3–23.3 | | 18.6–22.5 | 18.6–22.5 | 18.5–22.4 | 18.7–22.6 | 15.1–18.6 | 17.5–21.3 | 14.7–18.1 | 16. *M. paganus* |
